# Supplementary material for: Modelling Skylarks (Alauda arvensis) to Predict Impacts of Changes in Land Management and Policy: Development and Testing of an Agent-Based Model
Source: PLoS One. 2013 Jun 6;8(6):e65803. doi: 10.1371/journal.pone.0065803 (PMC3675089; doi:10.1371/journal.pone.0065803)
Supplement: Supporting Information S4 — The skylark ODdox as a zipped archive. (ZIP) [file pone.0065803.s004.zip › Skylark_ODdox/struct_low_pri_pair-members.html]

ALMaSS Skylark ODdox: Member List


ALMaSS Skylark ODdox
 2.0


- Main Page
- Related Pages
- Classes
- Files

- Class List
- Class Index
- Class Hierarchy
- Class Members

LowPriPair< ELEMTYPE > Member List

This is the complete list of members for LowPriPair< ELEMTYPE >, including all inherited members.

|  |  |  |
| --- | --- | --- |
| LowPriPair() | LowPriPair< ELEMTYPE > | inline |
| LowPriPair(const ELEMTYPE &a\_element, long a\_priority) | LowPriPair< ELEMTYPE > | inline |
| m\_element | LowPriPair< ELEMTYPE > |  |
| m\_pri | LowPriPair< ELEMTYPE > |  |
| operator<(const LowPriPair &a\_another\_pri\_pair) const | LowPriPair< ELEMTYPE > | inline |
| size\_type typedef | LowPriPair< ELEMTYPE > |  |
| value\_type typedef | LowPriPair< ELEMTYPE > |  |


- Generated on Thu Jan 10 2013 13:15:36 for ALMaSS Skylark ODdox by
   1.8.1.1
